# Single-electron Spin Resonance in a Quadruple Quantum Dot

**DOI:** 10.1038/srep31820

**Published:** 2016-08-23

**Authors:** Tomohiro Otsuka, Takashi Nakajima, Matthieu R. Delbecq, Shinichi Amaha, Jun Yoneda, Kenta Takeda, Giles Allison, Takumi Ito, Retsu Sugawara, Akito Noiri, Arne Ludwig, Andreas D. Wieck, Seigo Tarucha

**Affiliations:** 1 Center for Emergent Matter Science, RIKEN, 2-1 Hirosawa, Wako, Saitama 351-0198, Japan; 2 Department of Applied Physics, University of Tokyo, Bunkyo, Tokyo 113-8656, Japan; 3 Angewandte Festkörperphysik, Ruhr-Universität Bochum, D-44780 Bochum, Germany; 4 Quantum-Phase Electronics Center, University of Tokyo, Bunkyo, Tokyo 113-8656, Japan; 5 Institute for Nano Quantum Information Electronics, University of Tokyo, 4-6-1 Komaba, Meguro, Tokyo 153-8505, Japan

## Abstract

Electron spins in semiconductor quantum dots are good candidates of quantum bits for quantum information processing. Basic operations of the qubit have been realized in recent years: initialization, manipulation of single spins, two qubit entanglement operations, and readout. Now it becomes crucial to demonstrate scalability of this architecture by conducting spin operations on a scaled up system. Here, we demonstrate single-electron spin resonance in a quadruple quantum dot. A few-electron quadruple quantum dot is formed within a magnetic field gradient created by a micro-magnet. We oscillate the wave functions of the electrons in the quantum dots by applying microwave voltages and this induces electron spin resonance. The resonance energies of the four quantum dots are slightly different because of the stray field created by the micro-magnet and therefore frequency-resolved addressable control of each electron spin resonance is possible.

Electron spins in semiconductor quantum dots (QDs) have relatively long coherence times in solid state devices^1^,^2^,^3^,^4^ and potential scalability by utilizing current extensive semiconductor fabrication techniques. Considered good candidates for quantum bits^5^ in quantum information processing^6^,^7^, the required elementary operations on the spin-1/2 qubits for quantum information processing have been demonstrated recently. The spin states are initialized and read out using the Pauli spin blockade (PSB)^8^ or tunneling to the leads from Zeeman split energy levels^9^,^10^. Rotation of single spins has been realized by electron spin resonance (ESR)^11^. Addressability and the speed of single spin rotation are improved by micro-magnet (MM) induced ESR^12^,^13^. High-fidelity single-spin rotation decoupled from the fluctuating nuclear spin environment was demonstrated^14^. Entanglement operations of two spins are realized by utilizing exchange interaction and fast two qubit operations have been demonstrated^15^,^16^,^17^,^18^. This scheme for the spin-1/2 qubit is applicable to a wide variety of materials including Si, which has a long spin coherence time^3^,^4^.

Scale up of the QD system is crucial to realize larger scale quantum gate operations and also explore multi-spin physics. To this end, spin qubit experiments on multiple QDs have been reported in recent years. In triple QDs, PSB has been observed^19^,^20^ and the exchange only qubit utilizing a triple QD as a single qubit has been demonstrated^21^,^22^,^23^,^24^. Towards three spin-1/2 qubits^25^, ESR in a triple QD was recently realized^26^. Experiments on quadruple QDs (QQDs) have also been started^27^,^28^, and a QQD is utilized for realization of two qubit operations on singlet-triplet qubits^29^. For four spin-1/2 qubits, the precise charge state control in a tunnel coupled QQD has been demonstrated in the few-electron regime^30^.

In this paper, we demonstrate four distinctly addressable electron spin resonances in a QQD. First, we realize few-electron charge states in a QQD required to observe PSB. Second, we observe PSB for readout of ESR signals. The blocked triplet components are created by singlet-triplet mixing induced by the nuclear spins and the MM. Finally, we observe four ESR signals corresponding to the four individual spins in the QQD.

## Results

### Device and Charge states

**Figure 1:**
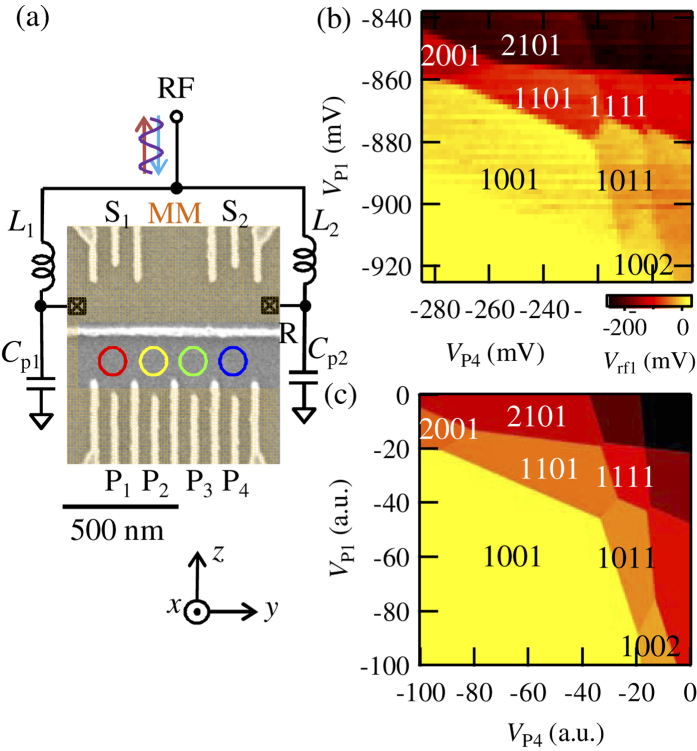
(**a**) Scanning electron micrograph of the device and the schematic of the measurement setup. A QQD is formed at the lower side and the charge states are probed by the charge sensor QDs at the upper side. The charge sensors are connected to resonators formed by the inductors *L*_1_ and *L*_2_ and the stray capacitances *C*_p1_ and *C*_p2_ for the RF reflectometry. A MM is deposited on the shaded region on the top of the device, which creates local magnetic fields to induce ESR. The external magnetic field is applied in plane along the *z* axis. (**b**) *V*_rf1_ as a function of *V*_P4_ and *V*_P1_. Changes of the charge states are observed. The number of the electrons in each QD is shown as *n*_1_, *n*_2_, *n*_3_, *n*_4_. (**c**) Calculated charge stability diagram of a QQD. The experimental result (**b**) is reproduced by considering the capacitively coupled QQD model. *n*_1_, *n*_2_, *n*_3_, *n*_4_ are shown in the figure.

Figure 1(a) shows a scanning electron micrograph of the device. By applying negative voltages on the gate electrodes, which appear light gray in the picture, a QQD and two QD charge sensors^31^ are formed at the lower and the upper sides, respectively. The QD charge sensors are connected to RF resonators formed by the inductors *L*_1_ and *L*_2_ and the stray capacitances *C*_p1_ and *C*_p2_ (resonance frequency *f*_res1_ = 298 MHz, *f*_res2_ = 207 MHz) for the RF reflectometry^31^,^32^,^33^. The number of electrons in each QD *n*_1_, *n*_2_, *n*_3_, and *n*_4_ is monitored by the intensity of the reflected RF signal *V*_rf1_ and *V*_rf2_. A change in the electrostatic environment around the sensing dots changes their conductance, shifts the tank circuit resonance and modifies *V*_rf1_ and *V*_rf2_ measured at *f*_res1_ and *f*_res2_. A MM is deposited on the shaded region on the top of the device, which creates local magnetic fields to induce ESR. The external magnetic field is applied in the plane along the *z* axis to induce Zeeman splitting and magnetizes the MM. The shape of the MM is specially designed to realize strong driving of the electron spin rotations by the large field gradient and splitting of the ESR frequencies by the Zeeman field differences between the dots^34^. Thanks to this MM, we can realize a stable magnetic field which is difficult to achieve by the fluctuating nuclear spins and the scheme is material independent.

Figure 1(b) is the charge stability diagram of the QQD. We measured *V*_rf1_ as a function of the plunger gate voltages of QD_4_ *V*_P4_ and QD_1_ *V*_P1_. We observe the change of *V*_rf1_, as the result of the charge states in the QQD. Charge transition lines with four different slopes are observed reflecting the different electrostatic coupling of the QQD to *V*_P4_ and *V*_P1_. *n*_1_, *n*_2_, *n*_3_, and *n*_4_ are assigned as shown in Fig. 1(b) by counting the number of charge transition lines from the fully depleted condition [*n*_1_, *n*_2_, *n*_3_, *n*_4_] = [0, 0, 0, 0]. Figure 1(c) shows the calculated charge state of the QQD. By considering the capacitively coupled QQD model^30^,^35^, we reproduce the observed charge stability diagram. We find the characteristic “goggle” structure, which is formed by the charge transition lines around [1, 1, 1, 1], [1, 1, 0, 1] and [1, 0, 1, 1] charge states. In the [1, 1, 1, 1] state, each dot contains a single electron and this state is useable as a four qubit system of the spin-1/2 qubit.

### Spin blockade

To readout the spin states of the qubits, PSB^8^ is a powerful tool. If the triplet spin states are formed in the neighboring QDs, the charge transition [1, 1, 0, 1] → [2, 0, 0, 1] ([1, 0, 1, 1] → [1, 0, 0, 2]) is forbidden because of Pauli exclusion principle. In the stability diagrams in Fig. 1(b,c), the spin blockade can be expected around the charge transition lines between [1, 1, 0, 1] and [2, 0, 0, 1], and between [1, 0, 1, 1] and [1, 0, 0, 2]. Note that charge boundaries between [1, 1, 1, 1] → [2, 0, 1, 1] and [1, 1, 1, 1] → [1, 1, 0, 2], which is required to observe PSB around the [1, 1, 1, 1] state, never coexist on a single *V*_P1_ − *V*_P4_ plane and we need to switch into *V*_P1_ − *V*_P2_ and *V*_P3_ − *V*_P4_ planes^30^. For experimental simplicity, we choose [1, 1, 0, 1] → [2, 0, 0, 1] and [1, 0, 1, 1] → [1, 0, 0, 2] boundaries to demonstrate PSB in this work.

**Figure 2:**
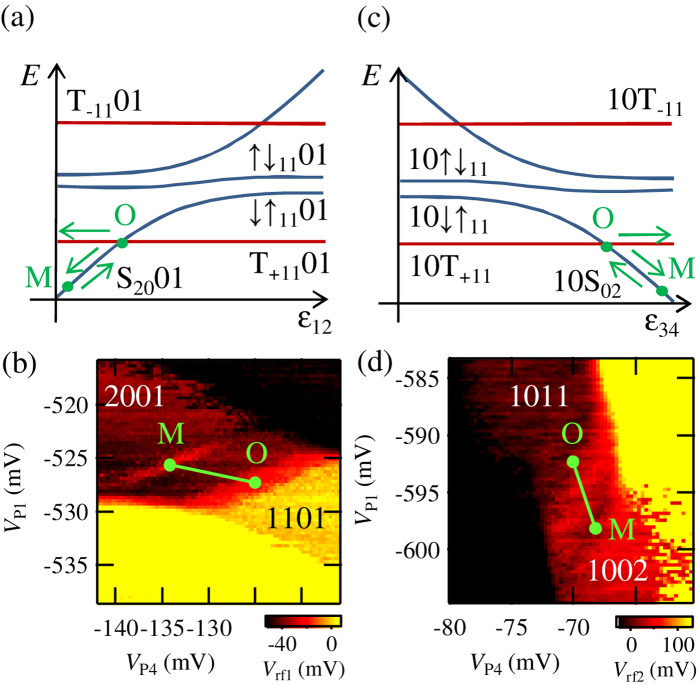
(**a,c**) Energy diagrams and schematics of the pulse operation to observe PSB in QD1 and QD2 (QD3 and QD4). The T_+11_01 (10T_+11_) component is populated at the operation point O by using the S_11_01 ⇔ T_+11_01 (10S_11_ ⇔ 10T_+11_) mixing. The triplet component is observed as the [1, 1, 0, 1] ([1, 0, 1, 1]) charge state at the measurement point M. (**b**,**d**) Observed *V*_rf1_ (*V*_rf2_) as a function of *V*_P4_ and *V*_P1_. The pulse sequences are indicated by lines in the figures. The change of *V*_rf1_ (*V*_rf2_) as the result of the spin blocked signals are observed around M.

We apply voltage pulses on *V*_P1_ and *V*_P4_ to observe spin blocked states. The operation schematics are shown in Fig. 2(a,c). We apply an external magnetic field of 0.5 T to induce Zeeman splitting. We start from the ground singlet state in QD_1_ S_20_01 (in QD_4_ 10S_02_). The triplet plus component T_+11_01 in QD_1_ and QD_2_ (10T_+11_ in QD_3_ and QD_4_) is populated at the operation point O by using the singlet-triplet mixing S_11_01 ⇔ T_+11_01 (10S_11_ ⇔ 10T_+11_) induced by the nuclear spins and the MM stray magnetic fields^36^. At the measurement point M, the triplet components stay in the [1, 1, 0, 1] ([1, 0, 1, 1]) charge state because of PSB and the singlet components relax to the [2, 0, 0, 1] ([1, 0, 0, 2]) charge state. Then, this blockade can be observed as the change of *V*_rf1_ (*V*_rf2_).

Figure 2(b,d) show the observed *V*_rf1_ (*V*_rf2_) as a function of *V*_P4_ and *V*_P1_. The operation pulses are cycled constantly at each point of the graph. We apply voltage pulses with fixed amplitudes as shown as lines in Fig. 2(b,d). The directions of the pulses on the stability diagrams are chosen to modulate the detuning, the energy difference of the levels between QD_1_ and QD_2_ (between QD_3_ and QD_4_). Note that we are also able to control QD_2_ and QD_3_ by *V*_P1_ and *V*_P4_ because of the finite capacitive coupling. Sensor 1 is used for Fig. 2(b) (Sensor 2 for Fig. 2(d)) to maximize the charge sensitivity. The changes of *V*_rf1_ (*V*_rf2_) are observed around M when the operation point O hits the singlet-triplet mixing point. These correspond to the spin blocked signals.

### Electron spin resonance

**Figure 3:**
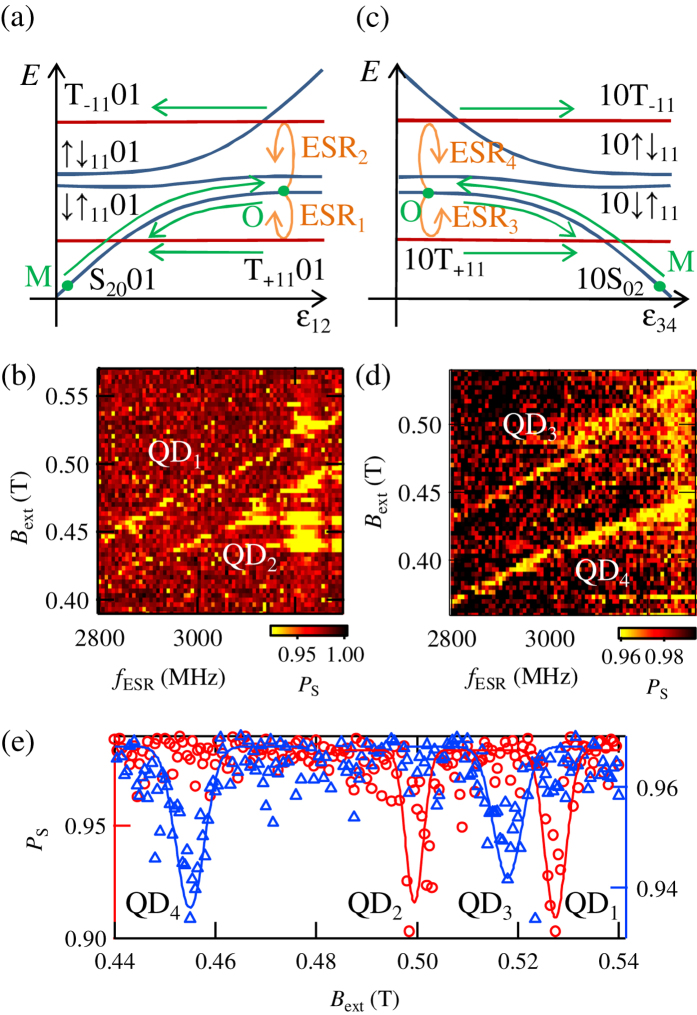
(**a,c**) Schematics of the energy diagrams and the pulse operations to observe ESR in QD1 and QD2 (QD3 and QD4). States are prepared as ↓↑_11_01 (10↓↑_11_) due to Δ*B*_*z*_ between QD_1_ and QD_2_ (QD_3_ and QD_4_) by the MM, which is larger than the singlet-triplet splitting at the operation point O. The states evolve into T_+11_01 or T_−11_01 (10T_+11_ or 10T_−11_) states by ESR. The created triplet components are observed as [1, 1, 0, 1] ([1, 0, 1, 1]) charge states at the measurement point M. (**b,d**) Observed *P*_S_ as a function of *f*_ESR_ and *B*_ext_. ESR occurs when the applied microwave frequency matches the external magnetic field plus the stray field created by the micro-magnet *hf*_ESR_ = *gμ*(*B*_ext_ + *B*_MMz_). (**e**) Observed *P*_S_ as a function of *B*_ext_ at *f*_ESR_ = 3265 MHz. Dips of *P*_S_ are observed when ESR occurs. The circles (triangles) show the results measured in QD1 and QD2 (QD3 and QD4). The traces are Gaussian eye guides.

Next, we apply a microwave voltage on gate R to induce ESR (with the frequency *f*_ESR_). The operation schematics are shown in Fig. 3(a,c). In the present device, the Zeeman field difference Δ*B*_*z*_ between QD_1_ and QD_2_ (QD_3_ and QD_4_) by the MM will be larger than the singlet-triplet splitting at the operation point O and the eigenstates are ↓↑_11_01 and ↑↓_11_01 (10↓↑_11_ and 10↑↓_11_), not S_11_01 and T_0 11_01 (10S_11_ and 10T_0 11_) (see Supplementary Information). We prepare the states ↓↑_11_01 in QD_1_ and QD_2_ (10↓↑_11_ in QD_3_ and QD_4_) by adiabatically pulsing from S_20_01 (10S_02_). Then, we apply microwaves at the operation point O. These applied microwaves create an oscillating electric field around the gate R and thus induce movements of the QD electron’s wave functions. These oscillations of the wave functions are converted into oscillating magnetic fields along the *x* axis perpendicular to the external magnetic field in the field gradient created by the MM and ESR is induced^12^,^13^. The triplet components T_+11_01 or T_−11_01 (10T_+11_ or 10T_−11_) are populated by ESR and detected as the [1, 1, 0, 1] ([1, 0, 1, 1]) charge states.

Figure 3(b,d) show the singlet return probability *P*_S_ as a function of *f*_ESR_ and the external magnetic field *B*_ext_. *P*_S_ is calculated from *V*_rf1_ (*V*_rf2_) by using the method reported in the refs 23,37. The measurement time is 30 *μ*s and set shorter than the relaxation time *T*_1_ > 100 *μ*s. We can see the decrease of *P*_S_ when the applied microwave frequency matches the external magnetic field plus the *z* component of the stray field created by the MM *hf*_ESR_ = *gμ*(*B*_ext_ + *B*_MMz_). The ESR dips of *P*_S_ are also observed in Fig. 3(e), which show *P*_S_ as a function of *B*_ext_ at *f*_ESR_ = 3265 MHz.

## Discussion

The slopes of the ESR lines in Fig. 3(b,d) give a value of the g-factor as |*g*| = 0.37 ± 0.03 that is consistent with reported values in previous experiments^38^,^39^,^40^.

We realize addressable control of the operation by choosing appropriate *B*_ext_ and *f*_ESR_ such that the separation of the ESR dips is larger than their width as in Fig. 3(e). From Fig. 3(e), the local Zeeman field differences between the quantum dots *B*_MMz12_, *B*_MMz13_, *B*_MMz14_ are evaluated as *B*_MMz12_ = 28 mT, *B*_MMz13_ = 9 mT, *B*_MMz14_ = 73 mT. If there is no misalignment of the QD positions, *B*_MMz12_ < *B*_MMz13_ < *B*_MMz14_ is expected from the design of the MM^34^. This discrepancy is attributed to the misalignment of the QD positions from the center of the MM. The observed values of the local Zeeman field are explained by shifts of the QD positions of around 100 nm in the *z* direction, which is possible in this QQD device (see Supplementary Information). The unexpected position-shift might be compensated by additional tuning of the gate voltages or removing inhomogeneous potentials by using undoped device structures^41^,^42^,^43^.

In conclusion, we have demonstrated formation of few-electron charge states, and observed spin blockade and four distinct ESR signals in a QQD. The four observed ESR dips are well separated and we are able to individually address spins by choosing the appropriate *B*_ext_ and *f*_ESR_. These results will be important for four or more spin-1/2 qubits, multiple qubit operations, and demonstration of larger scale quantum gate operations. These also contribute to exploring multi-spin physics in controlled artificial systems.

## Methods

### Device structure and measurement

The device was fabricated from a GaAs/AlGaAs heterostructure wafer with an electron sheet carrier density of 2.0 × 10^15^ m^−2^ and a mobility of 110 m^2^/Vs at 4.2 K, measured by Hall-effect in the van der Pauw geometry. The two-dimensional electron gas is formed 90 nm under the wafer surface. We patterned a mesa by wet-etching and formed Ti/Au Schottky surface gates by metal deposition, which appear light gray in Fig. 1(a). All measurements were conducted in a dilution fridge cryostat at a temperature of 13 mK.

## Additional Information

**How to cite this article**: Otsuka, T. *et al*. Single-electron Spin Resonance in a Quadruple Quantum Dot. *Sci. Rep*. **6**, 31820; doi: 10.1038/srep31820 (2016).

## Supplementary Material

Supplementary Information

## References

[b1] Bluhm H. . Dephasing time of GaAs electron-spin qubits coupled to a nuclear bath exceeding 200 *μ*s. Nat. Phys. 7, 109–113 (2011).

[b2] Shulman M. D. . Suppressing qubit dephasing using real-time Hamiltonian estimation. Nat. Commun. 5, 5156 (2014). 25295674 10.1038/ncomms6156 PMC4214408

[b3] Kawakami E. . Electrical control of a long-lived spin qubit in a Si/SiGe quantum dot. Nat. Nano. 9, 666–670 (2014). 10.1038/nnano.2014.153 25108810

[b4] Veldhorst M. . An addressable quantum dot qubit with fault-tolerant control-fidelity. Nat. Nano. 9, 981–985 (2014). 10.1038/nnano.2014.216 25305743

[b5] Loss D. & DiVincenzo D. P. Quantum computation with quantum dots. Phys. Rev. A 57, 120–126 (1998).

[b6] Nielsen M. A. & Chuang I. L. Quantum Computation and Quantum Information. (Cambridge University Press, 2000).

[b7] Ladd T. D. . Quantum computers. Nature 464, 45–53 (2010). 20203602 10.1038/nature08812

[b8] Ono K. . Current Rectification by Pauli Exclusion in a Weakly Coupled Double Quantum Dot System. Science 297, 1313–1317 (2002). 12142438 10.1126/science.1070958

[b9] Elzerman J. M. . Single-shot read-out of an individual electron spin in a quantum dot. Nature 430, 431–435 (2004). 15269762 10.1038/nature02693

[b10] Nowack K. C. . Single-Shot Correlations and Two-Qubit Gate of Solid-State Spins. Science 333, 1269–1272 (2011). 21817015 10.1126/science.1209524

[b11] Koppens F. H. L. . Driven coherent oscillations of a single electron spin in a quantum dot. Nature 442, 766–771 (2006). 16915280 10.1038/nature05065

[b12] Tokura Y. . Coherent Single Electron Spin Control in a Slanting Zeeman Field. Phys. Rev. Lett. 96, 047202 (2006). 16486882 10.1103/PhysRevLett.96.047202

[b13] Pioro-Ladriere M. . Electrically driven single-electron spin resonance in a slanting Zeeman field. Nat. Phys. 4, 776–779 (2008).

[b14] Yoneda J. . Fast Electrical Control of Single Electron Spins in Quantum Dots with Vanishing Influence from Nuclear Spins. Phys. Rev. Lett. 113, 267601 (2014). 25615383 10.1103/PhysRevLett.113.267601

[b15] Petta J. R. . Coherent Manipulation of Coupled Electron Spins in Semiconductor Quantum Dots. Science 309, 2180–2184 (2005). 16141370 10.1126/science.1116955

[b16] Brunner R. . Two-Qubit Gate of Combined Single-Spin Rotation and Interdot Spin Exchange in a Double Quantum Dot. Phys. Rev. Lett. 107, 146801 (2011). 22107226 10.1103/PhysRevLett.107.146801

[b17] Maune B. M. . Coherent singlet-triplet oscillations in a silicon-based double quantum dot Nature 481, 344–347 (2012). 22258613 10.1038/nature10707

[b18] Veldhorst M. . A two-qubit logic gate in silicon Nature 526, 410–414 (2015). 26436453 10.1038/nature15263

[b19] Kobayashi T. . Cooperative Lifting of Spin Blockade in a Three-Terminal Triple Quantum Dot. arXiv:1311.6582 (2013).

[b20] Amaha S. . Two- and Three-Electron Pauli Spin Blockade in Series-Coupled Triple Quantum Dots. Phys. Rev. Lett. 110, 016803 (2013). 23383822 10.1103/PhysRevLett.110.016803

[b21] Laird E. A. . Coherent spin manipulation in an exchange-only qubit Phys. Rev. B 82, 075403 (2010).

[b22] Gaudreau L. . Coherent control of three-spin states in a triple quantum dot Nature Physics 8, 54–58 (2012).

[b23] Medford J. . Self-consistent measurement and state tomography of an exchange-only spin qubit Nature Nanotech 8, 654–659 (2013). 10.1038/nnano.2013.168 23995458

[b24] Eng K. . Science Advances. 1, e1500214 (2015). 26601186 10.1126/sciadv.1500214 PMC4640653

[b25] Takakura T. . Triple quantum dot device designed for three spin qubits Appl. Phys. Lett. 97, 212104 (2010).

[b26] Noiri A. . Coherent electron-spin-resonance manipulation of three individual spins in a triple quantum dot. Appl. Phys. Lett 108, 153101 (2016).

[b27] Thalineau R. . A few-electron quadruple quantum dot in a closed loop. Appl. Phys. Lett. 101, 103102 (2012).

[b28] Takakura T. . Single to quadruple quantum dots with tunable tunnel couplings. Appl. Phys. Lett. 104, 113109 (2014).

[b29] Shulman M. D. . Demonstration of Entanglement of Electrostatically Coupled Singlet-Triplet Qubits Science 336, 202–205 (2012). 22499942 10.1126/science.1217692

[b30] Delbecq M. R. . Full control of quadruple quantum dot circuit charge states in the single electron regime. Appl. Phys. Lett. 104, 183111 (2014).

[b31] Barthel C. . Fast sensing of double-dot charge arrangement and spin state with a radio-frequency sensor quantum dot. Phys. Rev. B 81, 161308 (2010).

[b32] Schoelkopf R. J. . The Radio-Frequency Single-Electron Transistor (RF-SET): A Fast and Ultrasensitive Electrometer. Science 280, 1238–1242 (1998). 9596572 10.1126/science.280.5367.1238

[b33] Reilly D. J., Marcus C. M., Hanson M. P. & Gossard A. C. Fast single-charge sensing with a rf quantum point contact. Appl. Phys. Lett. 91, 162101 (2007).

[b34] Yoneda J. . Robust micromagnet design for fast electrical manipulations of single spins in quantum dots. Appl. Phys. Exp. 8, 084401 (2015).

[b35] van der Wiel W. G. . Electron transport through double quantum dots. Rev. Mod. Phys. 75, 1–22 (2002).

[b36] Chesi S. . Single-spin manipulation in a double quantum dot in the field of a micromagnet. Phys. Rev. B 90, 235311 (2014).

[b37] Barthel C., Reilly D. J., Marcus C. M., Hanson M. P. & Gossard A. C. Rapid Single-Shot Measurement of a Singlet-Triplet Qubit. Phys. Rev. Lett. 103, 160503 (2009). 19905680 10.1103/PhysRevLett.103.160503

[b38] Potok R. M. . Spin and Polarized Current from Coulomb Blockaded Quantum Dots. Phys. Rev. Lett. 91, 016802 (2003). 12906563 10.1103/PhysRevLett.91.016802

[b39] Hanson R. . Zeeman Energy and Spin Relaxation in a One-Electron Quantum Dot. Phys. Rev. Lett. 91, 196802 (2003). 14611599 10.1103/PhysRevLett.91.196802

[b40] van Beveren L. H. W. . Spin filling of a quantum dot derived from excited-state spectroscopy. New J. Phys. 7, 182 (2005).

[b41] Borselli M. G. . Pauli spin blockade in undoped Si/SiGe two-electron double quantum dots. Appl. Phys. Lett. 99, 063109 (2011).

[b42] See A. M. . Impact of Small-Angle Scattering on Ballistic Transport in Quantum Dots. Phys. Rev. Lett. 108, 196807 (2012). 23003076 10.1103/PhysRevLett.108.196807

[b43] MacLeod S. J. . Hybrid architecture for shallow accumulation mode AlGaAs/GaAs heterostructures with epitaxial gates. Appl. Phys. Lett. 106, 012105 (2015).

