# Supplementary material for: Single-electron Spin Resonance in a Quadruple Quantum Dot
Source: Sci Rep. 2016 Aug 23;6:31820. doi: 10.1038/srep31820 (PMC4994114; doi:10.1038/srep31820)
Supplement: Supplementary Information [file srep31820-s1.pdf]

Supplemental Material to 'Single-electron Spin Resonance in a Quadruple Quantum Dot'

Tomohiro Otsuka,[1,2] Takashi Nakajima,[1,2] Matthieu R. Delbecq,[1,2] Shinichi Amaha,[1]
Jun Yoneda,[1,2] Kenta Takeda,[1,2] Giles Allison,[1] Takumi Ito,[1,2] Retsu Sugawara,[1,2]
Akito Noiri,[1,2] Arne Ludwig,[3] Andreas D. Wieck,[3] and Seigo Tarucha[1,2,4,5]

[1]*Center for Emergent Matter Science, RIKEN,*
2-1 Hirosawa, Wako, Saitama 351-0198, Japan
[2]*Department of Applied Physics, University of Tokyo, Bunkyo, Tokyo 113-8656, Japan*
[3]*Angewandte Festkörperphysik, Ruhr-Universität Bochum, D-44780 Bochum, Germany*
[4]*Quantum-Phase Electronics Center, University of Tokyo, Bunkyo, Tokyo 113-8656, Japan*
[5]*Institute for Nano Quantum Information Electronics,*
University of Tokyo, 4-6-1 Komaba, Meguro, Tokyo 153-8505, Japan

(Dated: July 13, 2016)

MAGNETIC FIELDS CREATED BY THE MICRO-MAGNET

Figures S1 (a) and (b) show the calculated slanting magnetic field $\partial B_{\mathrm{M}x}/\partial z$ ((a)) and the Zeeman field $B_{\mathrm{M}z}$ ((b)) created by the micro-magnet (MM) on the quantum dot (QD) layer in the device. The calculation was performed using the MATHEMATICA RADIA package available at http://www.esrf.fr/. In Figs. S1 (a) and (b), the origin ($y = 0, z = 0$) is set at the center of the MM gap. $\partial B_{\mathrm{M}x}/\partial z$ is used to produce the electron spin resonance (ESR) combined with the movement of the quantum dot (QD) position in the z direction induced by applied microwave voltages. The shape of the MM is specially tailored to produce large values of $\partial B_{\mathrm{M}x}/\partial z$ over a larger area to make the device robust against misalignment of the QD and the MM layers, which might be as large as 100 nm in real devices [1]. Fast Rabi oscillations exceeding 100 MHz have been reported utilizing a similar MM [2]. The typical value of the slanting field is $\partial B_{\mathrm{M}x}/\partial z \approx 0.8$ mT/nm around positions of the QDs.

The spatial change of $B_{\mathrm{M}z}$ creates a difference in the Zeeman field at each position of the QQD. $B_{\mathrm{M}z}$ shifts the value of the external magnetic field at the center of the ESR peak ($hf = g\mu(B_{\mathrm{ext}z} + B_{\mathrm{M}z})$). Well separated ESR peaks enable addressable control of the spins in the QQD.

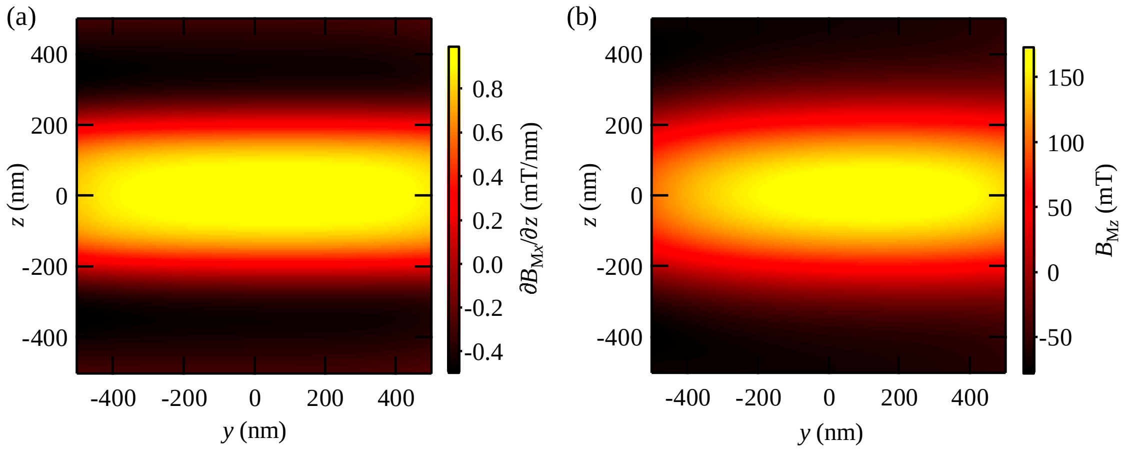

FIG. S1: (a) Calculated $\partial B_{\mathrm{M}x}/\partial z$ created by the MM on the QD layer of the device. Typically the value of the slanting field is $\partial B_{\mathrm{M}x}/\partial z \approx 0.8$ mT/nm around positions of the QDs. (b) Calculated B_z. The inhomogeneous $B_{\mathrm{M}z}$ creates separation of ESR peaks of the QQD.

ESTIMATED POSITION OF THE QUADRUPLE QUANTUM DOT

Figure S2 shows the spatial distribution of $B_{\mathrm{M}z}$ and one possible position for each of the QDs in the QQD which can explain the observed ESR peak separations in Figs. 3 (b), (d) and (e). The lines show the position of the gates when we assume the alignment of the QD layer and the MM layer is perfect. The circles show the estimated positions of the QDs. This arrangement of the QDs is possible because of the impurity potentials, asymmetric gate voltages and misalignment of the layers in real devices, which shift the QD positions. The gate voltages in this experiment are tuned to optimize the tunneling barriers for the spin blockade measurements. The unexpected position-shift might be compensated by additional tuning of the gate voltages or removing inhomogeneous potentials by using undoped device structures [3–5].

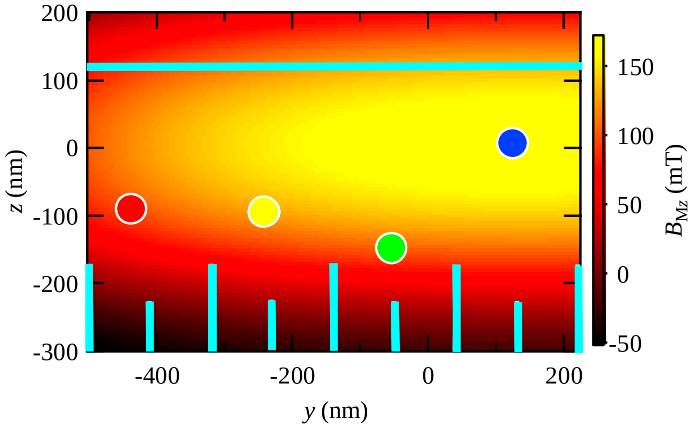

FIG. S2: Spatial distribution of $B_{\mathrm{M}z}$ and the estimated position of the QQD. The lines and circles show the positions of the gates and the estimated positions of the QDs.

---

[1] Yoneda, J. *et al*. Robust micromagnet design for fast electrical manipulations of single spins in quantum dots. *Appl. Phys. Exp.* **8**, 084401 (2015).

[2] Yoneda, J. *et al*. Fast Electrical Control of Single Electron Spins in Quantum Dots with Vanishing Influence from Nuclear Spins. *Phys. Rev. Lett.* **113**, 267601 (2014).

[3] Borselli, M. G. *et al*. Pauli spin blockade in undoped Si/SiGe two-electron double quantum dots. *Appl. Phys. Lett.* **99**, 063109 (2011).

[4] See, A. M. *et al*. Impact of Small-Angle Scattering on Ballistic Transport in Quantum Dots. *Phys. Rev. Lett.* **108**, 196807 (2012).

[5] MacLeod, S. J. *et al*. Hybrid architecture for shallow accumulation mode AlGaAs/GaAs heterostructures with epitaxial gates. *Appl. Phys. Lett.* **106**, 012105 (2015).
